# HOW DO CLINICIANS USE, EXPERIENCE, AND VALUE APPLICATIONS OF OUTCOME INFORMATION IN DAILY CARE? A MIXED-METHODS STUDY

**DOI:** 10.2340/jrm.v57.42610

**Published:** 2025-03-19

**Authors:** Yara VAN KOOIJ, Nina LOOS, Grada ARENDS, Kasia TABEAU, Harm SLIJPER, Joris VELTKAMP, Ruud SELLES, Robbert WOUTERS, on behalf of OUTCOME-BASED HEALTHCARE RESEARCH GROUP

**Affiliations:** 1 Department of Rehabilitation Medicine, Erasmus MC, Rotterdam, The Netherlands; 2 Department of Plastic, Reconstructive, and Hand Surgery, Erasmus MC, Rotterdam, The Netherlands; 3 Xpert Handtherapie, Utrecht, The Netherlands; 4 Erasmus School of Health Policy and Management, Erasmus University Rotterdam, Rotterdam, The Netherlands; 5 Equipe Zorgbedrijven, Eindhoven, The Netherlands

**Keywords:** patient-centred care, patient-reported outcome measures, value-based healthcare, health dashboards, patient outcome information assessments

## Abstract

**Objective:**

To support data-driven healthcare, digital applications of patient and outcome information bundled in dashboards can be used in daily care. This study investigated the usage, user-friendliness, and added value of patient and outcome information applications from a clinician’s perspective.

**Design:**

We used a mixed-methods design, including surveys (*n* = 56 clinicians), interviews (*n* = 16 clinicians), and eye-tracking experiments (*n* = 8 clinicians) across 3 different settings: a specialized clinic, a rehabilitation centre, and a general hospital. The applications, bundled in dashboards, include visual representations of patient information, individual treatment goals, screening tools for mental health, pain, and physical function, individual predictions of recovery and treatment effect, visuals of treatment outcome information, and identification of extreme values that fall outside the expected values.

**Results:**

Applications were used for managing patient expectations, treatment selection, goal setting, and treatment evaluation. While usage frequency varied between applications and clinicians generally reported positive experiences with outcome information, a complex interaction of factors influenced use in clinical practice. The value of each application depends on its clinical actionability and clinicians’ confidence.

**Conclusion:**

From clinicians’ perspectives, the applications provide meaningful conversation starters, can lead to a more targeted conversation, and allow for better patient–clinician connection.

## LAY ABSTRACT

Collecting information on patients and their outcomes is becoming more popular. However, the systems we have now do not always give easy-to-understand information that fits with the work routines of clinicians. This study delves into how clinicians view the use of digital tools displaying patient and outcome information in their daily work. Our findings reveal that clinicians find these tools helpful in discussing patient goals, managing expectations, and evaluating treatments. While many clinicians appreciate their potential, the usefulness of these tools depends on the clinical actionability of the information they provide. Some clinicians have concerns regarding these digital tools potentially overlooking nuances and the patient’s contextual situation. This study highlights the importance of these digital aids in fostering meaningful conversations between clinicians and patients, ultimately improving the quality of healthcare interactions.

Driven by the growing interest in value-based healthcare, growing emphasis is placed on routinely measuring outcomes in daily clinical care (1). As a result, routine patient and outcome information is increasingly collected, and using this outcome information can be beneficial to healthcare processes such as clinician–patient communication and treatment monitoring (2). Patient reported outcome measures (PROMs) play a key role in this process, providing direct insights into patients’ health status and serving as a frequently used tool for understanding treatment effectiveness. However, using this information on an individual patient level in daily clinical care remains challenging (3–7). Simply making this information available in the electronic health record is usually insufficient to provide value to patients and clinicians (8). This highlights the need for an adequate infrastructure that provides intuitive and practical insights for clinicians and patients while seamlessly fitting into the clinicians’ daily workflows (3, 8, 9). Digital tools that summarize the patient and outcome information may provide such insights (8, 10). These tools could streamline the integration of outcome information at the individual patient level by providing clinicians with real-time, accessible insights.

In recent years, we collaborated with clinicians and patients to develop applications of outcome information to support healthcare processes (hereafter referred to as “applications”). The applications include (*i*) visuals of general patient information, (*ii*) the display of the personal request for help and individual treatment goals, (*iii*) screening tools for physical and mental health, (*iv*) individual predictions of recovery and treatment outcomes, (*v*) visuals of outcome information, and (*vi*) identification and feedback of extreme values (i.e., scores outside the expected range). These applications are integrated into a dashboard accessible to clinicians via electronic patient records. Hypothetically, integrating these applications into daily clinical care can empower clinicians to use patient and outcome information at the individual patient level. Ultimately, this integration might enhance shared decision-making and improve expectation management (8, 10–14).

While previous studies have explored clinicians’ perspectives on PROMs and dashboards (4, 10, 11, 13–18), little is known about how clinicians use these applications during consultations and integrate them into their workflows. Gaining more profound insights into these aspects and understanding how the applications impact decision-making, expectation management, and treatment evaluation is needed to optimize data-driven and personalized healthcare strategies. Therefore, this study aimed to understand the clinicians’ use of patient and outcome information applications during daily clinical care. Specifically, we addressed the following research questions:

1. To what extent do clinicians use the applications?
2. How do clinicians use the applications?
3. What is the usability of the applications according to clinicians, and what factors affect the usability?
4. How do clinicians value the applications, and what factors affect the perceived value?

## METHODS

### Study design

This is a convergent mixed-methods study reported according to the Mixed Methods Article Reporting Standards (19), comprising quantitative and qualitative data through surveys, interviews, and eye tracking. This combination of methods provided a thorough understanding of behaviours and motivations related to dashboard usage. All methods were given equal priority, and we synthesized all data using a pre-defined data triangulation method to answer our research questions.

### Study setting

Data were collected between March 2022 and September 2022 at Xpert Clinics (a specialized outpatient clinic for hand and wrist care), the neurology department of a general hospital (Onze Lieve Vrouwe Gasthuis, OLVG), and Rijndam Rehabilitation (a rehabilitation centre). We chose these 3 to represent both elective and non-elective healthcare settings. The study was approved by the medical ethics review board of Erasmus MC, and all participants provided informed consent.

### Participants

We included clinicians treating patients across 3 settings: hand and wrist disorders at Xpert Clinics, chronic pain at Rijndam Rehabilitation, and stroke care at OLVG. Surveys and eye tracking were conducted with clinicians from Xpert Clinics (hand surgeons, *n* = 27; therapists, *n* = 137) and Rijndam Rehabilitation (rehabilitation physicians, *n* = 3; psychologists, *n* = 2). Surveys were sent to all clinicians with access to the dashboards at Xpert Clinics and Rijndam. The surveys and eye tracking focused on actual use during the daily clinic. Therefore, we did not include clinicians from OLVG, as their dashboards were still in the pilot testing phase with limited user engagement. Purposive sampling for eye tracking included clinicians with diverse roles and attitudes toward dashboard use, with no additional inclusion or exclusion criteria. For the semi-structured interviews, we used purposive sampling with maximum variation. We included clinicians from all 3 settings with diverse occupations (medical and allied health professionals), ages, and experiences. Clinicians were contacted by phone or email by the researchers. Both early adopters of the applications and more critical clinicians were approached to ensure a range of perspectives.

### Applications of patient and outcome information

Each centre developed customized dashboards and applications tailored to clinicians’ and patients’ needs derived from their daily clinical care workflows. Detailed dashboard examples are provided in Figs S1–S3. For OLVG, only individual predictions of recovery and treatment effects, assessed through a stand-alone application, were included in this study.

*Visuals of general patient information.* At Xpert Clinics and Rijndam Rehabilitation, patients complete a questionnaire before their initial appointment. The local dashboards display information from this questionnaire, such as relevant medical history, medication use, body mass index, and type of work.

*Personal request for help and individual treatment goals.* At Xpert Clinics, the Patient Specific Needs evaluation (PSN) is used before the initial appointment. This patient-reported tool identifies and evaluates individual information needs, treatment goals, and improvement goals to support decision-making and expectation management (20). At Rijndam Rehabilitation, patients complete a questionnaire regarding their request for help, selecting from predefined options (e.g., “to find the cause” or “accept the pain”).

*Screening tools on mental health, pain, and function.* At Xpert Clinics and Rijndam Rehabilitation, screening tools are employed before the initial appointment to serve as a conversation starter and support expectation management and decision-making. At Xpert Clinics, the Ultra-Short Mental Health Screening tool is used, which provides valid insights into pain catastrophizing, psychological distress, and illness perception, using only 4 questions (21). Additionally, a Numeric Rating Scale (NRS) is used for pain and hand function. At Rijndam Rehabilitation, mental health is assessed using validated PROMs: the Pain Catastrophizing Scale, the Psychological Inflexibility in Pain Scale, and the Pain Self-Efficacy Questionnaire.

*Individual predictions of recovery and treatment effect.* Prediction models are available at Xpert Clinics to predict individual recovery and treatment outcomes (22, 23). These models, which use patient-specific characteristics and baseline PROM scores as input variables, predict the probability of improving at least the minimally important change of 2 points on the 0–10 NRS for pain and function (24). OLVG offers predictions of recovery after stroke by presenting individualized graphs that display the expected progression of the arm and hand function (25). No prediction models are available at Rijndam Rehabilitation.

*Visuals of outcome information.* Xpert Clinics follows the International Consortium for Health Outcomes Measurement standard for hand and wrist conditions (7). Throughout the treatment course, patients complete PROMs to assess their health status over time, and clinicians measure their strength and range of motion. At Rijndam Rehabilitation, the Hospital Anxiety and Depression Scale, 12-Item Short Form Survey, Pain Disability Index, and Canadian Occupational Performance Measure are administered at multiple time points during the rehabilitation process to assess patient progress. The PROM data and corresponding norm values are displayed in dashboards using graphs and tables, enabling clinicians to assess treatment progress.

*Identification and feedback of extreme values.* Extreme values are values deviating from the expected range based on patient information, values that leave little room for improvement (e.g., perfect hand function at baseline), or risk factors for poor recovery (e.g., pain catastrophizing behaviour). Xpert Clinics and Rijndam Rehabilitation use colour coding to highlight such values in the pain, function, and mental health screening tools (e.g., red for pain catastrophizing behaviour, green for normal scores). Additionally, text boxes highlight situations with little room for improvement. Colour coding is also applied to the visuals of outcome information over time, enabling clinicians to track whether a patient improves or worsens during rehabilitation.

### Survey

We developed a survey to investigate the extent to which and how clinicians use the applications of outcome information. This survey also evaluated the user-friendliness and perceived value of the applications while also collecting sociodemographic characteristics of clinicians (e.g., age, clinical role, and years of experience).

### Eye tracking

We used eye tracking to assess the extent to which clinicians use the applications and how they use them. By tracking the clinicians’ gaze behaviour and eye movements on the dashboards (26), we measured when and how long their gaze lingered on each application (27).

We performed eye tracking in a lab setting with fictional cases and in a daily clinic setting during actual patient consultations to ensure various patient cases were assessed. A Tobii X3-120 eye tracking bar was mounted on a desktop computer screen for the lab sessions. The eye tracking bar collected gaze data at a sample rate of 120 Hz (Tobii Pro, Danderyd, Sweden). The eye tracking bar was calibrated before the start of the experiment for each clinician by looking at 9 predefined targets on the screen (9-point 3x3 calibration) (26). Seven fictional cases were presented to clinicians within the dashboard (Table SI). After reading basic information on the case (e.g., symptoms and medical history), clinicians consulted the dashboard and were asked to provide a treatment proposal while thinking aloud. For the daily clinic sessions, we used Tobii Glasses 3 eye tracking glasses with a sampling rate of 50 Hz (Tobii Pro, Danderyd, Sweden) (27). Clinicians conducted patient consultations as usual without specific instructions on application use. The lab and daily clinic sessions were video and audio-recorded (28). Fixation times (i.e., the total viewing time) per application were calculated from the data using RNotebook (28).

### Interviews

We conducted semi-structured interviews with clinicians to assess their perceived application use, factors influencing usability, and perceived value. YvK, a physical therapist trained in qualitative research with an MSc in clinical health sciences, conducted all interviews. YvK is an experienced user and co-developer of the applications and an expert in routine outcome measurement. The interview guide was based on the research aims. Each interview was recorded and then transcribed using Amber script (www.amberscript.com).

### Data analyses

We performed descriptive statistics on the survey questions using the median with interquartile range. Eye-tracking results from the lab setting were visualized in heatmaps using the iMotions software (https://imotions.com/) (28). In addition, 4 authors (Yvk, NL, JV, RW) watched the eye-tracking recordings from the lab and the daily clinic sessions. They monitored how often each application was used, whether the clinician discussed it with the patient, whether it affected treatment decisions, or the conversation with the patient.

Interviews and analyses were alternatively conducted, allowing insights from earlier interviews to inform and refine subsequent ones, leading to more focused and targeted questions. To ensure the validity of the findings, we conducted member checks by sending a summary to all participants and asking if they agreed with the interpretations. We used a descriptive design with conventional content analysis, meaning that the coding categories are derived directly from the text data (29). Content analysis is commonly used in mixed-methods studies because it aligns closely with the terminology used in interviews and research questions (29–32). YvK analyzed all interviews using MAXQDA (VERBI Software, 2021; MAXQDA - VERBI Software GmbH, Berlin, Germany). To validate and ensure the accuracy of the coding process, a second researcher (DA) independently co-coded the first 5 interviews. The analysis process began by highlighting all text relevant to our research questions. We then defined categories and discussed how the codes related to each other until YvK and DA reached a consensus on the main categories and definitions.

### Data triangulation

We used a triangulation protocol to synthesize the quantitative and qualitative findings (33). As a first step, we performed convergent coding, which included comparing the survey results, eye tracking, and interviews for an initial answer to the research questions based on that specific data collection method (i.e., only the survey, eye tracking, or interviews). The second step included the convergence assessment: the research team discussed the results of the previous steps. This was done in multiple meetings, allowing the researchers to return to the raw data from the survey, eye tracking, and interviews if there was disagreement between the findings of each data collection method, and to discuss specific interpretation issues. The final step was a complete comparison, combining findings from the survey, eye tracking, and interviews to answer the research questions.

## RESULTS

### Participants

Fifty-six of the 173 invited clinicians completed the survey: 11 hand surgeons, 42 hand therapists, 2 rehabilitation physicians, and 1 psychologist (Table I). Three clinicians participated in the lab setting eye tracking, and 5 clinicians with 15 patients in the daily clinic eye tracking. We conducted 16 semi-structured interviews involving 10 clinicians from Xpert Clinics, 4 from Rijndam Rehabilitation, and 3 from OLVG (Table II). The interviews lasted 30–45 min. After 16 interviews, we reached theoretical sufficiency as no new concepts emerged in subsequent interviews (34).

**Table I:** Demographic characteristics of the survey participants (*n* = 56)

Sex, *n* (%)	
Male	22 (39)
Female	33 (59)
Rather not say	1 (2)
Age, *n* (%)	
> 45 years	4 (7)
18–35 years	36 (64)
36–45 years	16 (29)
Organization, *n* (%)	
Xpert Clinics	53 (95)
Rijndam Rehabilitation	3 (5)
Function, *n* (%)	
Hand surgeon	11 (20)
Physical therapist	26 (46)
Occupational therapist	16 (19)
Psychologist	1 (2)
Rehabilitation physician	2 (4)
Work experience in years, mean (SD)	6.6 (6.2)

**Table II:** Demographic characteristics of the interview participants (*n* = 16)

Participant	Age	Sex	Function	Organization	Work experience in years
C 1	36–45	Male	Surgeon	Xpert Clinics	6 up to 10
C 2	36–45	Male	Surgeon	Xpert Clinics	0 up to 5
C 3	36–45	Male	Surgeon	Xpert Clinics	6 up to 10
C 4	25–35	Female	Physical therapist	Xpert Clinics	0 up to 5
C 5	25–35	Female	Occupational therapist	Xpert Clinics	6 up to 10
C 6	36–45	Male	Physical therapist	Xpert Clinics	> 10
C 7	25–35	Male	Physical therapist	Xpert Clinics	0 up to 5
C 8	> 46	Male	Surgeon	Xpert Clinics	> 10
C 9	36–45	Male	Surgeon	Xpert Clinics	6 up to 10
C 10	25–35	Female	Rehabilitation physician	Rijndam Rehabilitation	6 up to 10
C 11	36–45	Female	Psychologist	Rijndam Rehabilitation	> 10
C 12	25–35	Male	Rehabilitation physician	Rijndam Rehabilitation	0 up to 5
C 13	> 46	Female	Physician	OLVG^a^	> 10
C 14	> 46	Male	Nurse	OLVG	> 10
C 15	36–45	Female	Occupational therapist	OLVG	> 10
C 16	36–45	Male	Rehabilitation physician	Rijndam Rehabilitation	6 up to 10

a OLVG: Onze Lieve Vrouwe Gasthuis.

### To what extent are clinicians using patient and outcome information applications?

**Fig. 1:**
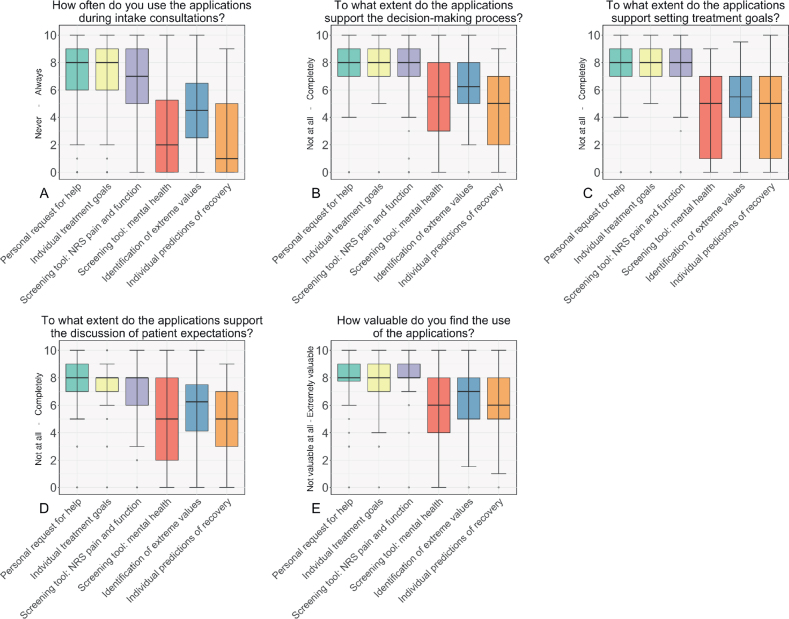
Clinicians’ perspectives on using patient and outcome information before the intake consultation with patients (*n* = 53). The figure shows the survey answers to the following questions: (A) How often do you use the following applications during intake consultations with patients? [0 = Never, 10 = Always]. (B) To what extent do the following applications support the decision-making process during intake consultations with patients? [0 = To no extent, 10 = To a large extent]. (C) To what extent do the following applications support setting treatment goals for patients during intake consultation with patients? [0 = To no extent, 10 = To a large extent]. (D) To what extent do the following applications support the discussion of the expectations of patients during intake consultations with patients? [0 = To no extent, 10 = To a large extent]. (E) How valuable do you find the use of the following applications during intake consultations with patients? [0 = Not valuable at all, 10 = Very valuable]. The bold, black horizontal line represents the median score. PROMs: patient reported outcome measures; CROMs: clinician reported outcome measures. The question on satisfaction with the applications was not asked for the identification of extreme values because this was considered part of the screening tools for mental health and NRS pain and function. The expected time until return to work was considered part of the individual predictions.

The survey and eye-tracking data showed a wide variation in the frequency of use between the clinicians and applications (Table III). The personal request for help and individual treatment goals were commonly addressed during intake sessions. These applications had a median (interquartile range [IQR]) score of 8.0 (6.0–9.0) on the survey (range 0–10, 10 = always, see Fig. 1). At follow-up sessions, these applications were used less frequently. Additionally, the screening tools for pain and function were often used during intake, with a median survey score of 7.0 (5.0 to 8.0, see Fig. 1).

**Table III:** Triangulation table for the findings from the survey, eye tracking, and interviews

Research questions	Associated survey question	Associated results of survey questions (median [IQR])^a^ (*n* = 53 clinicians)	Associated eye tracking findings from a clinical setting (*n* = 3 clinicians with 16 patients)	Associated eye tracking findings from lab session (*n* = 3 clinicians with 7 cases)	Associated findings from the interviews (*n* = 16 clinicians)	Conclusion following syntheses of the data
To what extent are clinicians using the applications?	How often do you use the applications during the preparation on the first appointment with your patients [0 = Never, 10 = Always]?	5.0 [2.0, 8.0]	In preparation for the first appointment, clinicians look most frequently at the patient information (6/16), request for help and treatment goal (6/16), and the screener for pain and function (5/16) During the first appointment, clinicians mostly use the NRS pain and function screener (3/16) During a follow-up appointment, they also use outcome information over time (3/16) Other applications are infrequently used during consultation	During the first appointment, clinicians frequently look at the patient’s information, the personal request for help, and the individual treatment goal At follow-up, the heatmap shows that clinicians look after the treatment progress using the outcome information	NA^b^	The survey and eye-tracking data show wide variation in the frequency of use between participants and between applications
	How often do you use the applications during the first appointment with your patients [0 = Never, 10 = Always]?	5.0 [1.0, 8.0]				
	How often do you use the applications during follow-up appointments with your patients [0 = Never, 10 = Always]?	5.0 [1.0, 7.0]				
How do clinicians use the applications?	To what extent do the applications support the decision-making process during first consultations with patients [0 = To no extent, 10 = To a large extent]?	7.0 [5.0, 8.0]	NA	Clinicians use the applications to inform patients about treatment options, support decision-making on treatment choices, discuss expectations and evaluate the treatment progress	Clinicians use the application to interact with the patient, discuss expectations, inform patients about treatment options, evaluate treatment progress, and communicate between clinicians	All 3 methods suggest that applications can be supportive in the interaction between clinician and patient; to discuss expectations, for treatment selection and goal setting, and during treatment evaluation
	To what extent do the applications support setting treatment goals for patients during first consultation with patients [0 = To no extent, 10 = To a large extent]?	7.0 [4.0, 8.0]				
	To what extent do the applications support discussing the expectations of patients during first consultations with patients [0 = To no extent, 10 = To a large extent]?	7.0 [5.0, 8.0]				
	To what extent do the applications support the evaluation of the treatment during follow-up consultations with patients [0 = To no extent, 10 = To a large extent]?	7.0 [4.0, 8.0]				
	To what extent do the applications support discussing the expectations of patients during follow-up consultations with patients [0 = To no extent, 10 = To a large extent]?	6.0 [4.0, 8.0]				
How user friendly do find the clinicians the applications and what factors affect the usability?	How well do you generally understand the following applications [0 = Not at all, 10 = Very well]?	8.0 [7.0, 10]	NA	NA	Practical factors influence usability: e.g., the degree of knowledge a clinician has about the applications, the adequacy of data availability, and the extent to which usage fits into the daily workflow Sufficient time is a prerequisite	The survey and interviews suggest that the applications are user-friendly The perceived time investment varies between clinicians
	How satisfied are you with the use of the applications during first consultations with patients [0 = Very unsatisfied, 10 = Very satisfied]?	8.0 [5.0, 8.0]				
	How satisfied are you with the use of the applications during follow-up consultations with patients [0 = Very unsatisfied, 10 = Very satisfied]?	6.0 [5.0, 8.0]				
	In general, does the use of the applications cost you extra time or does it save time [Costs extra time – Neutral – Saves me time]?	57% costs extra time, 6% saves time, and 47% neither saves nor costs time				
How do clinicians value the applications and what factors affect the perceived value?	How valuable do you find the use of the applications during first consultations with patients [0 = Not valuable at all, 10 = Very valuable]?	7.5 [5.0, 8.0]	NA	NA	The perceived value depends on practical factors: e.g., the loss of personal (eye) contact, the diagnosis, the course of rehabilitation and the questionnaires) and personal factors, e.g., the experience of the clinician and the amount of confidence they have in the applications	The survey and interviews show that clinicians find the applications valuable The variation of the perceived value depends on practical and more personal factors
	How valuable do you find the use of the applications during follow-up consultations with patients [0 = Not valuable at all, 10 = Very valuable]?	7.0 [3.75, 8.0]				

a IQR: interquartile range,

b NA: not applicable.

**Fig. 2:**
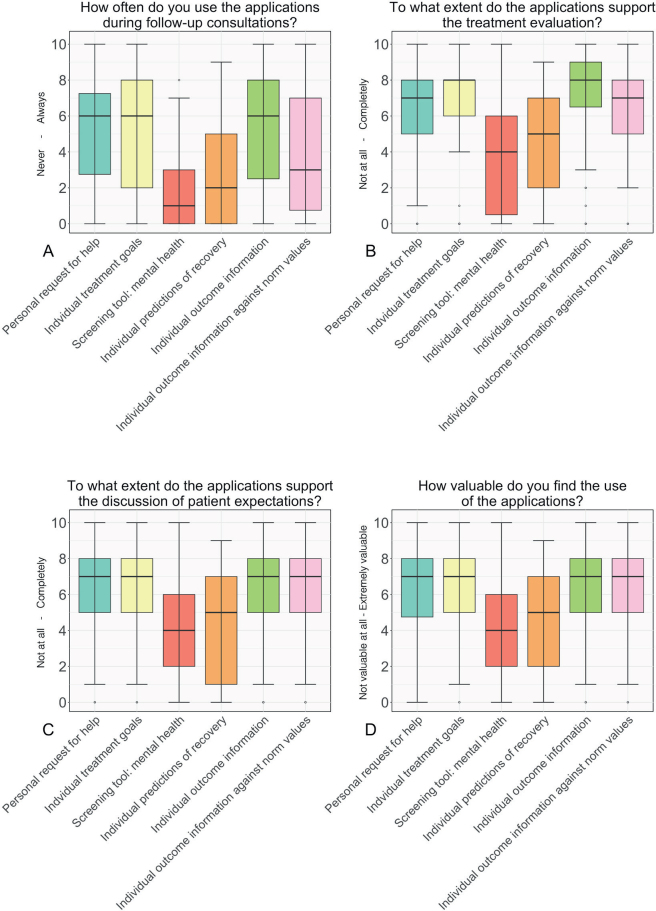
Clinicians’ perspectives on using patient and outcome information during the follow-up consultation with patients (*n* = 53). The figure shows survey answers to the questions: (A) How often do you use the following applications during follow-up consultations with patients? [0 = Never, 10 = Always]. (B) To what extent do the following applications support the treatment evaluation during follow-up consultations with patients? [0 = To no extent, 10 = To a large extent]. (C) To what extent do the following applications support the discussion of the expectations of patients during intake consultations with patients? [0 = To no extent, 10 = To a large extent]. (D) How valuable do you find the use of the following applications during follow-up consultations with patients? [0 = Not valuable at all, 10 = Very valuable]. The bold, black horizontal line represents the median score. PROMs: patient reported outcome measures, CROMs: clinician reported outcome measures.

**Fig. 3:**
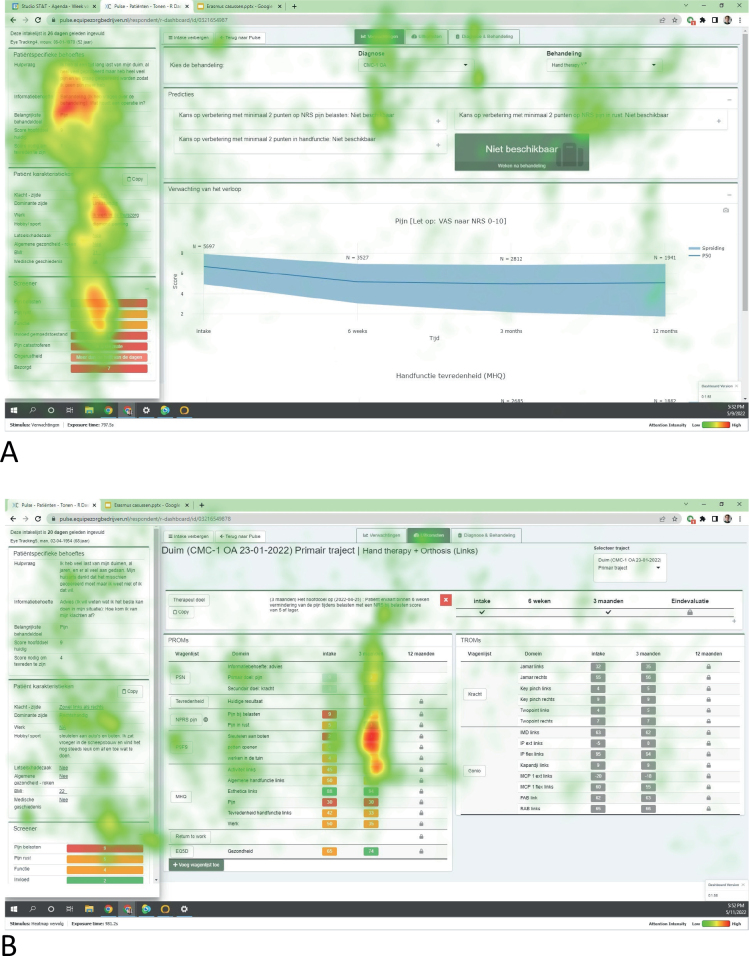
Heatmaps of the dashboard, including the applications with the combined viewing behaviours of 3 surgeons during the eye tracking sessions in lab setting with fictive patients. (A) viewing behaviour for fictive patients at intake, and (B) viewing behaviour for fictive patients at follow-up consultations. The coloured areas indicate areas that the surgeons looked at, with the different colours representing how much the areas were looked at (red = looked at a lot, green = looked at a little). The heatmaps show that clinicians mostly looked at the personal request for help and treatment goals, patient information such as medical history and occupation status, and the screener for pain, function, and mental health. During follow-up consultations, clinicians also looked at PROM data over time.

The eye-tracking heatmaps were aligned: clinicians mostly looked at the personal request for help and individual treatment goals, patient information visuals, and the pain and function screening tools (Fig. 3). The survey and eye tracking indicated less frequent use of the mental health screening tool, prediction models, and the visuals of outcome information over time (Figs 1–2).

### How do clinicians use the applications?

The applications can support the interactions between clinician and patient across various stages of daily care, including expectation management, decision-making, and treatment evaluation (Table III). These processes were supported mainly by personal requests for help, individual treatment goals, and screening tools for pain and function (Fig. 1). This is reflected by one of the clinicians:

If that’s your request for help, I will not improve that with surgery…. In the end, that patient is grateful to me for saying that. (C3)

Furthermore, clinicians used the applications in decision-making scenarios, particularly in cases where surgery may not be advisable. As one clinician noted:

If someone has very little pain, you might create more pain with surgery. Then you’re more inclined not to do that. (C3)

The applications are also used to evaluate treatment progress and to comfort, motivate, or assist patients in accepting and managing their symptoms, particularly during lengthy rehabilitation programmes or after disappointing outcomes. One clinician noted:

And you see … much better mobility. Yeah, that’s just nice for people. That confirms … their commitment. (C4)

### How user-friendly are the applications, and what factors affect their usability?

**Fig. 4:**
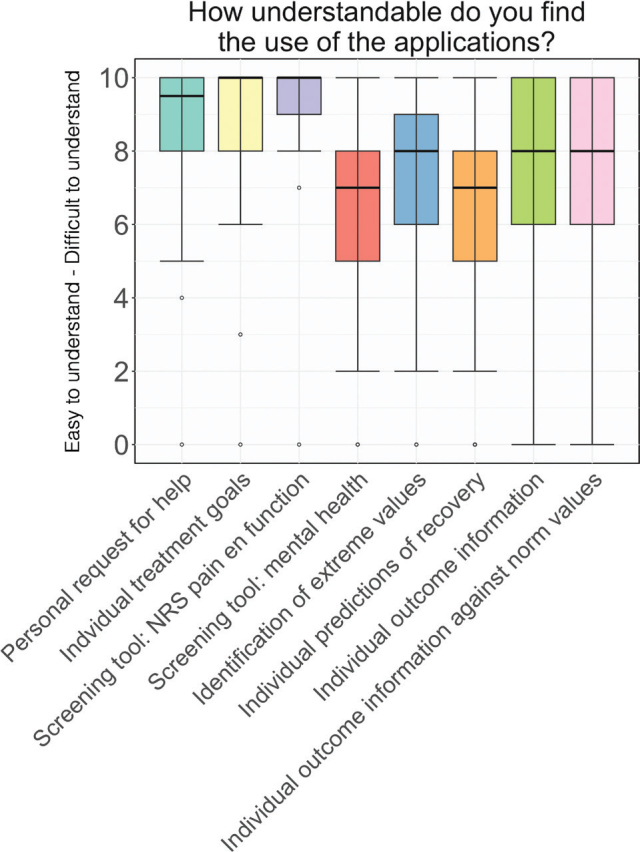
Ease of use. The general understandability of the applications. All applications scored a median of 7 or higher (range: 0–10) on ease of use.

**Fig. 5:**
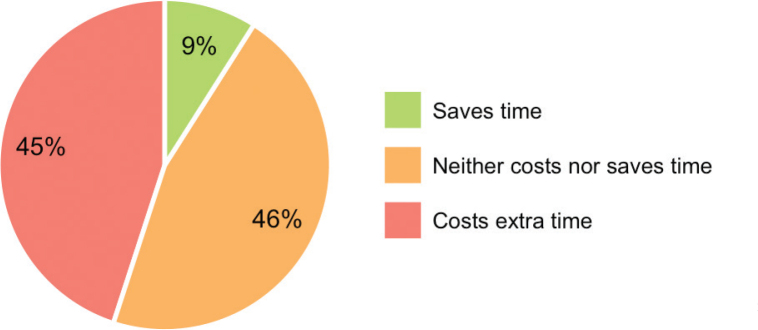
Time investment. Perceived time investment of clinicians using the applications.

Clinicians found the applications user-friendly (Table III). All applications scored a median of 7 or higher (range 0–10, 10 = difficult) on ease of use (Fig. 4). The perceived user-friendliness depended on the user’s knowledge of the applications, whether the information was available at the right time (e.g., readily accessible during patient consultations), and whether the applications fit the workflow. The perceived time investment varied widely (Fig. 5); some found it time-saving, enabling more efficient conversations and quicker identification of patients’ needs, while others considered it an administrative burden at the expense of time they could have spent with their patients. As one clinician expressed it:

In that fifteen minutes, I already have to tell everything, do everything and build a bond … this just doesn’t fit into that. (C8)

### How do clinicians value the applications, and what factors affect the perceived value?

Clinicians valued the personal request for help (median 8, 7.75–9, range 0–10, 10 = extremely valuable), individual treatment goals (median 8, 7–9), and pain and function screening tools (median 8, 8–9) highly. In contrast, they deemed other applications, such as the screening tool for mental health, extreme value detection, individual predictions, and outcome information over time, less valuable (Figs 1–2). The interviews highlight that the individual patient situation influences the perceived value of the applications (Table III). For example, while the mental health screener scored as less valuable in the survey, it can still benefit clinicians in specific circumstances, especially when notable values are identified. One clinician illustrates this:

If you’re struggling with a problem, and you’ve filled all that out in that questionnaire and … someone says: you’re worried a lot, aren’t you? … I find that of enormous added value. (C9)

The same applies to the individual predictions. These applications are valuable for managing unrealistic expectations but become less relevant when the expectations of the patient and clinician are already aligned. Furthermore, the applications were less relevant for minor hand and wrist surgery procedures, where clinicians felt they had sufficient experience and knowledge to educate patients adequately. As one clinician explained,

For example, with a trigger finger release … then I don’t see the added value. Except, of course, if it’s complicated, then you do start looking at it. (C5)

In addition, various clinician-related factors affected the perceived value of the applications. For instance, several clinicians indicated that displaying patient and outcome information on a computer screen disrupts eye contact, potentially impacting patient–clinician interaction and making integration into conversations more challenging. Furthermore, some clinicians struggled to trust the prediction model and emphasized that they prefer to make decisions with the patient instead of solely relying on the application. Some clinicians feel the prediction models account insufficiently for nuances and the patient’s contextual situation. One clinician articulated this sentiment, stating:

”A prediction model does not see the patient. It can only see numbers. (C8)”

## DISCUSSION

Although patient and outcome information is increasingly collected in routine healthcare, using this information on an individual patient level in daily care remains challenging (3, 4, 7). We examined how clinicians use outcome-based applications to support value-based healthcare and gathered their perspectives on its implementation across different clinical settings. Clinicians generally had positive experiences with patient and outcome information, regardless of differences in clinical setting, clinical role, or patient population. The applications were meaningful conversation starters, leading to a more targeted conversation and a better connection between patients and clinicians.

Our findings align with previous studies highlighting the advantages of integrating outcome information in routine care for expectation management, decision-making, and treatment evaluation (4, 11, 13, 15–17, 35–37). Some previous studies suggest that clinicians rely primarily on their clinical judgement and not on PROM data (12, 35), while our study indicates that clinicians did use PROM data in shared decision-making. This difference in findings might be because those studies present patients’ PROM scores as feedback, whereas, in our study, these scores are visualized within applications. Additionally, it may reflect the evolving healthcare landscape, where technological advancements and innovative solutions have increasingly integrated data into routine clinical workflows, enhancing accessibility and usability.

The use of eye tracking in our study was difficult to compare with other studies, as this novel approach has not been used in similar research. While eye tracking was only a minor component of our overall study, the results align closely with those from the surveys and interviews, reinforcing the robustness of our overall findings. This also highlights the potential of eye tracking as a valuable method for exploring the use of applications in clinical settings.

Although clinicians generally have positive experiences with patient and outcome information, applications in clinical practice are shaped by a complex interplay of factors, including patient-dependent factors. Clinicians are more likely to consult individual predictions concerning recovery and treatment effects when their expectations differ from the patients’, but are less likely to do so when their expectations align. Similarly, the mental health screening tool was particularly useful for communication when extreme values were detected. This suggests that the value of each application is dependent on the clinical actionability of the application’s output. Understanding which factors drive this actionability could further improve applications of outcome information and, thereby, value-based healthcare.

Personality traits and personal views of clinicians also affect the use of the applications. Some clinicians hesitate to rely on applications because they fear it could compromise the holistic perspective of healthcare, or they need help to trust these tools. Based on these findings, we recommend actively involving hesitant users in the design process to address potential variations in opinions to maximize trust and value for all clinicians.

This study has several limitations. Despite our sampling strategy, we may have primarily reached clinicians who are enthusiastic about using patient and outcome information. The study population’s young age may also have influenced the results, as younger individuals typically have a more favourable view of technical solutions and applications. Additionally, not all eligible clinicians completed our survey, and we are unaware of the underlying reason for this. However, our results show a wide range of responses, and clinicians in the interviews were also critical, suggesting that we achieved the desired variation in clinician perspectives. While standardized questionnaires, such as the System Usability Scale (38), are available for evaluating applications, we asked more specific questions regarding how healthcare providers use the applications in their daily practice. As a result, our study cannot be directly compared to others using standardized questionnaires.

A final limitation is that the survey was predominantly conducted at a specialized clinic for hand and wrist conditions. Our findings apply to settings where value-based healthcare is part of the organization’s strategy. Future studies may investigate the views of clinicians from other settings to investigate how they can make the transition towards value-based healthcare.

In conclusion, clinicians generally have positive experiences with patient and outcome information, regardless of differences in clinical settings, clinical roles, or patient populations. Personal requests for help, individual treatment goals, and screening tools were mainly used and valued. These applications help facilitate focused conversations, the discussion of treatment expectations, shared decision-making, goal setting, and treatment evaluation.

## Supplementary Material




