# Supplementary material for: HOW DO CLINICIANS USE, EXPERIENCE, AND VALUE APPLICATIONS OF OUTCOME INFORMATION IN DAILY CARE? A MIXED-METHODS STUDY
Source: J Rehabil Med. 2025 Mar 19;57:42610. doi: 10.2340/jrm.v57.42610 (PMC11980948; doi:10.2340/jrm.v57.42610)
Supplement: Supplementary file 1 [file JRM-57-42610-s1.pdf]

Supplementary material has been published as submitted. It has not been copyedited, or typeset by Journal of Rehabilitation Medicine

Table SI. Description of seven fictional patients with information on medical history and physical examination. The assignment for clinicians was as follows: use the medical history data and the information on the dashboard, make your choice of treatment, speak your choice aloud, and explain why as if the patient were sitting with you in the consulting room.

Number	Description of the fictional patient and medical history
**1**	A 46-year-old right-handed woman was referred to you by her general practitioner with the following referral: trigger finger right middle finger for which injection three months ago. After intensive gardening five months ago, she suddenly developed complaints about the right middle finger. First, a period of rest was prescribed by the general practitioner. When this gave insufficient relief, he gave an injection, from which the patient benefited for several weeks. Now recurrent symptoms.
**2**	A 59-year-old right-handed man has been experiencing pain in his left thumb base joint for over a year. He was referred to you by his general practitioner due to the severity of the pain.<br>For over a year, the patient has had complaints in his left thumb without apparent reason. According to his general practitioner, it is osteoarthritis, and nothing can be done. He now suffers a lot of pain in his daily activities, such as peeling apples, pulling up his pants, lifting plates, and working as a carpenter. He is limited in these activities by the pain.
**3**	A 58-year-old right-handed woman with osteoarthritis of the right thumb basal joint was referred by the general practitioner after completing hand therapy and splinting with limited effect.<br>For four months, she experienced complaints of the right thumb without any reason. She had an extensive course of hand therapy with exercises and a splint. This helped somewhat, but not much.
**4**	A 52-year-old left-handed woman came to the consultation referred by the general practitioner for thumb base osteoarthritis on the right side. She works in home care.<br>The patient has longstanding problems with her thumb and other joints, such as her knees. Before she was referred, she had a long course of hand therapy and splinting. This has helped her to function better in some daily situations, but she finds the pain too severe. She also worries about whether she can continue working in home care.

**5**	The general practitioner referred a 68-year-old man with the following question: thumb base osteoarthritis left with insufficient effect of hand therapy, is surgery an option? Initially, you chose to start hand therapy again, and the patient now comes to your consultation three months after start hand therapy.<br>The patient worked in the shipbuilding industry for years suffering from pain in both thumbs (on the left side much more than on the right side). Has already had extensive therapy and used several splints. The patient doubts whether he should have surgery; several people around him have told him that it has not helped them, and he wonders whether it will help him.
**6**	A 55-year-old right-handed man arrives at the 3-month check-up after a proximal row carpectomy on the right side due to radiocarpal osteoarthritis. The surgery proceeded without complications. Before the consultation, you learn from the hand therapist that the patient is not yet satisfied.
**7**	A 38-years-woman comes for consultation. During the consultation, it was noted that it is very likely to be a Triangular Fibrocartilage Complex injury on the right side: symptoms occurred after a fall on the outstretched hand. From the physical examination, the ulnar fovea remains painful on palpation. The distal radio-ulnar joint is more unstable on the right than on the left; the ulnar column test is positive. Otherwise, there are no abnormalities.

Table SII. Themes, findings, and quotes from the interviews with clinicians. In brackets, the number of clinicians where this theme was mentioned during the interview.

**How do clinicians use the applications?**		
Theme	*Findings (Number of interviews in which this theme was discussed)*	*Citation*
**Interaction**	1.1 Focused and in-depth conversation<br><br>(10/16)	1.1 “If you you're struggling with a problem, and you've filled all that out in that questionnaire and [..] someone says: you're worried a lot, aren't you? How annoying. [...] If you start with that in your consultation [...] I find that of enormous added value.” (C9)
	1.2 Responding to the patient's request for help<br><br>(8/16)	1.2 “If that's your request for help, I will not make that better with surgery. [...] In the end, that patient is grateful to me for saying that.”(C3)
	1.3 Dashboard provides background information<br><br>(12/16)	1.3 “Yes, and that just gives me an impression of how the patient functions. So, when I see a low score, I think I need to pay attention.” (C13)
	1.4 Visual objectification with dashboard<br><br>(11/16)	1.4 “Yes, a dashboard [...] because, at a glance, you can see how someone is doing.” (C11)
**Discuss expectations**	2.1 Discuss expectations<br><br>(11/16)	2.1 “And you can also discuss that with patients. You can expect this in six months because about 50 percent of all patients are still tired.” (C14)
**Treatment decision**	3.1 Support to inform patients<br><br>(6/16)	3.1 “Yes, [...] we've been doing that for years. Based on scientific articles [...] the same goes for the real live data.” (C2)

	3.2 Support in treatment choice and goals<br><br>(10/16)	3.2.1 “Suppose the depression and anxiety score is very high, which is a contraindication for our pain program. In that case, I also tune out those expectations [...] First, this is going to be treated because that's rehabilitation overarching [...]. People understand that right away.” (C10)
		3.2.2 “It takes away a whole branch of making decisions together [...] I'm going to look at a computer and use a prediction model to see what I should do next.” (C9)
		3.2.3 “Everyone has a different perception of pain [...]. If someone has very little pain, then you might create more pain with surgery. Then you're more inclined not to do that.” (C3)
		3.2.4 “I don't need to convince people of anything, but I like that they can form opinions based on information [...]. Because then you talk about the same thing.” (C15)
**Evaluation**	4.1 Evaluation, not often used, but valuable<br><br>(14/16)	4.1 “Yes, at least you do have a complete picture of: how is it going now and how was it before?” (C4)
	4.2 Added value: disappointing results or long-term recovery<br><br>(6/16)	4.2 “Those figures do help. Often you hear someone say, I don't see the difference, [...], and then you look at the figures, and they say, oh yes, it has improved quite a bit.” (C5)

	4.3 Added value: accepting and handling complaints<br><br>(12/16)	4.3 “You can try so hard [...] but look, this is what it is, and it's not getting any better.” (C6)
	4.4 Added value: motivate patients<br><br>(7/16)	4.4 “And you see [...] a lot better mobility. Yeah, that's just nice for people. That kind of confirms their commitment.” (C4)
	4.5 Added value: reassure patients<br><br>(8/16)	4.5 “Well, then you can reassure them; this is normal. This is what I expected.” (C3)
**Communication between clinicians**	5.1 Communication between clinicians<br><br>(5/16)	5.1 “Yes, intercollegially [...] It is also helpful to know immediately what the outcomes are and what is being worked towards because then you can tell the same story.” (C15)

**How user friendly do find the clinicians the applications and what factors affect the usability?**		
Theme	*Findings (Number of interviews in which this theme was discussed)*	*Citation*
**Usefulness**	6.1 The dashboard shows how the patient is doing.<br><br>(12/16)	6.1 “Yes, a dashboard allows you more because now you can see how someone is developing at a glance.” (C11)
	6.2 Prerequisite: knowledge of the applications<br><br>(10/16)	6.2 “Yes, but maybe if you have more knowledge of it, it does add value [...] But I don't see that now.” (C7)
	6.3 Prerequisite: data available at the right time<br><br>(14/16)	6.3.1 “So, when it's filled out, I look at it, but it's also often not filled out.” (C1)
		6.3.2 “If I [...] don't know if the data is available that I can or can't back up my

		story with, then I'm not going to use it.” (C2)
	6.4. Prerequisite: fitting into the workflow of daily practice<br><br>(11/16)	6.4 “No, not at all. Or, in this case, I have to start thinking very much: oh yes, shit, what should be where and how and how? And meanwhile, I feel that it all doesn't fit within that time.” (C6)
**Time investment**	7.1 Saves time; quicker response to complaint<br><br>(8/16)	7.1 “I also think we can use the short time we have [...] much more usefully by zooming in on what the patient needs.” (C13)
	7.2 Costs time; it is an administrative burden<br><br>(16/16)	7.2 “And in that fifteen minutes, I already have to tell everything, do everything and build a bond [...], this just doesn't fit into that.” (C8)

**What factors affect the perceived value?**		
Theme	*Findings (Number of interviews in which this theme was discussed)*	*Citation*
**Practical factors**	8.1 Using a dashboard can cause loss of personal (eye) contact<br><br>(8/16)	8.1 “You also have limited time in treatment. So then you make priorities, and then I prefer talking to a patient [...], rather than looking at the numbers” (C7)
	8.2 Added value: depends on diagnosis and course<br><br>(7/16)	8.2 “I don't actually miss it in my treatment, [...] for example with a trigger finger release [...], then I don't see the added value. Except of course if it's complicated, then you do start looking at it.” (C5)
	8.3 Added value: depending on questionnaires and applications	8.3.1 “Using those comprehensive questionnaires for your final consult, no, I don't see that” (C7)

	(3/16)	8.3.2 “That one is going very well, then is it misfilled?” (C4)
**Personal factors**	9.1 Prerequisite: confidence in applications (9/16)	9.1.1 “If those models are 100 percent conclusive, that would be interesting. But believing that, I find that rather difficult. [...] It's too variable [...] to trust in that.” (C7)
		9.1.2 “No, because a prediction model does not see the patient, it can only see numbers.” (C8)
	9.2 System should not determine treatment, but support (8/16)	9.2.1 “No, I think that the therapeutic view is still needed […]. Because otherwise the model would be even more complicated.” (C15)
		9.2.2 “I just don't believe that, that you have to get that out of a model. Surely that is also just gut feeling [...]. There has to be room for that.” (C9)
	9.3 Knowledge already present through conversation with patient (7/16)	9.3 “If you see someone once or twice a week […], that would be a little weird if you wouldn't realize something wasn't going right. I don't need a form for that.” (C6)
	9.4 Conversation is a tool to achieve something (5/16)	9.4 “So by asking questions in a conversation that patient comes to insights. I think if the patient fills out forms at home behind the laptop that he/she does not come to those insights.” (C6)
	9.5 Added value: depends on clinicians' experience	9.5 “I just need that data to make my story stronger, because of course I know the story. I do know from experience

	(5/16)	and from the literature what is the best treatment for that patient" (C2)
	9.6 Numbers don't say it all, conversation provides context<br><br>(3/16)	9.6.1 "So the lists and the dashboard gives a piece of indication; 'this may be influential,' but the conversation makes to see, how big is it really?" (C16)
		9.6.2 "Is it a pain score nine at intense use, once a month or a nine at rest, daily? Is someone distraught with pain? Those are the words that are helpful to me." (C8)
	9.7 Prefer no inside information<br><br>(2/16)	9.7 "Because then you are going to steer a little more into the conversation instead of leaving it open." (C5)
