# Supplementary material for: HOW DO CLINICIANS USE, EXPERIENCE, AND VALUE APPLICATIONS OF OUTCOME INFORMATION IN DAILY CARE? A MIXED-METHODS STUDY
Source: J Rehabil Med. 2025 Mar 19;57:42610. doi: 10.2340/jrm.v57.42610 (PMC11980948; doi:10.2340/jrm.v57.42610)
Supplement: Supplementary file 2 [file JRM-57-42610-s2.pdf]

Supplementary material has been published as submitted. It has not been copyedited, or typeset by Journal of Rehabilitation Medicine

Patientinfo

Naam	[redacted]
Leeftijd	[redacted]
Geslacht	Vrouw
BMI	20

Hoe is de pijn ontstaan?	Geleidelijk in de loop van maanden

Hulpvraag

Intake Alle antwoorden							
Dat de oorzaak van de pijn wordt gevonden	Dat de pijn minder erg wordt of overgaat	Minder medicijnen tegen de pijn nodig heb	Actiever worden ondanks mijn pijn	Mijn lichamelijke conditie verbeteren	Leren omgaan met mijn pijn	Accepteren dat ik pijn heb	Anders
Ja	Ja	Ja	Ja	Ja	Ja	Ja	1

Screening

	Screening psychologie en fysiotherapie Alle antwoorden	12 weken Alle antwoorden	3 mnd na uitbehandeling Alle antwoorden
PCS score	52	26	0
Normscore	score duidt op pijn catastroferen	score duidt op pijn catastroferen	score duidt niet op pijn catastroferen
Piekeren (max. 16)	16	8	0
Uitvergroten (max.12)	12	6	0
Hulpeloosheid (max. 24)	24	12	0

	Screening psychologie en fysiotherapie Alle antwoorden	12 weken Alle antwoorden
PIPS score	7	3.19
Normscore	hoog	beneden gemiddeld
Vermijding	7	3.2
Cognitieve fusie	7	3.17

	Screening psychologie en fysiotherapie Alle antwoorden	12 weken Alle antwoorden
PSEQ score (range 0-60)	0	30
Normscore	score duidt op een lage eigen-effectiviteit verwachting t.a.v. pijn	score duidt niet op een lage eigen-effectiviteit verwachting t.a.v. pijn

NRS

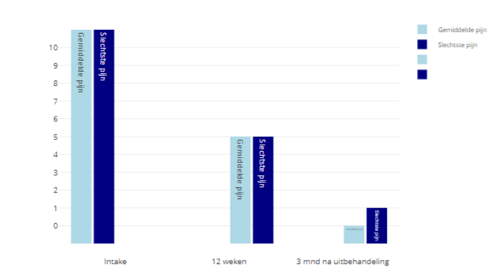

PDI

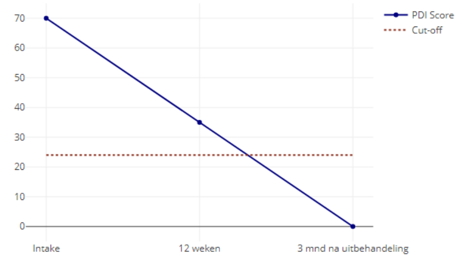

HADS

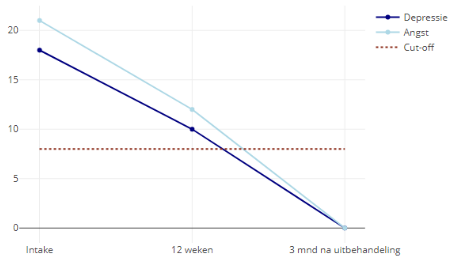

SF-12

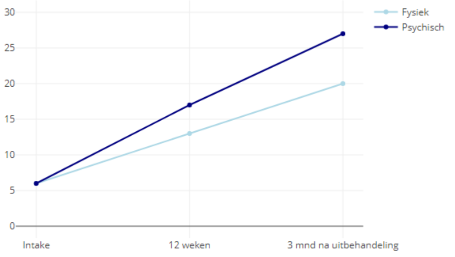

COPM

	Intake Alle antwoorden		
	Probleem	Uitvoering momenteel	Tevredenheid momenteel
1	Werken	1	1
2	Reizen	1	1
3	Hardlopen	1	1
4	Wielrennen	1	1
5	Piano spelen	1	1

12 weken Alle antwoorden		
Probleem	Uitvoering momenteel	Tevredenheid momenteel
Werken	10	10
Reizen	10	10
Hardlopen	10	10
Wielrennen	10	10
Piano spelen	10	10

Your measured and predicted arm/hand function

Your current arm/hand function score is 8. For 95% of patients in your situation, this score will be between 12 and 45 in 6 months.

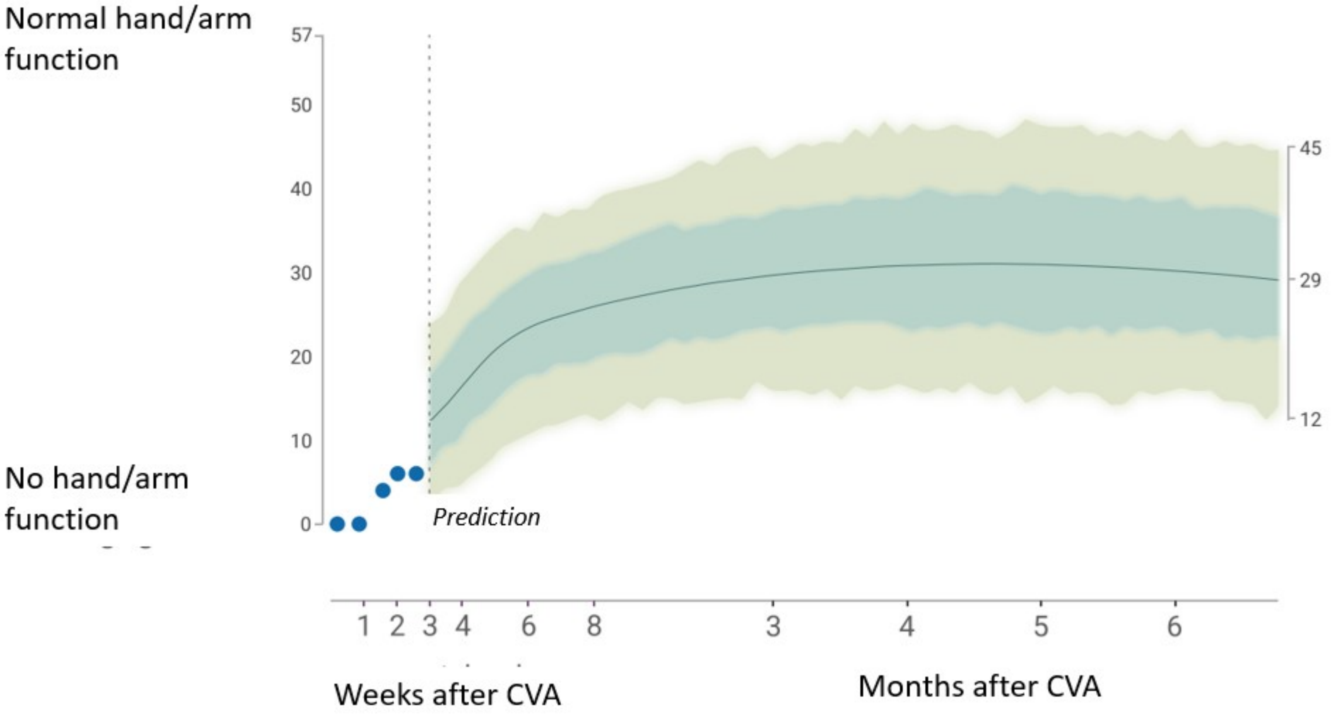

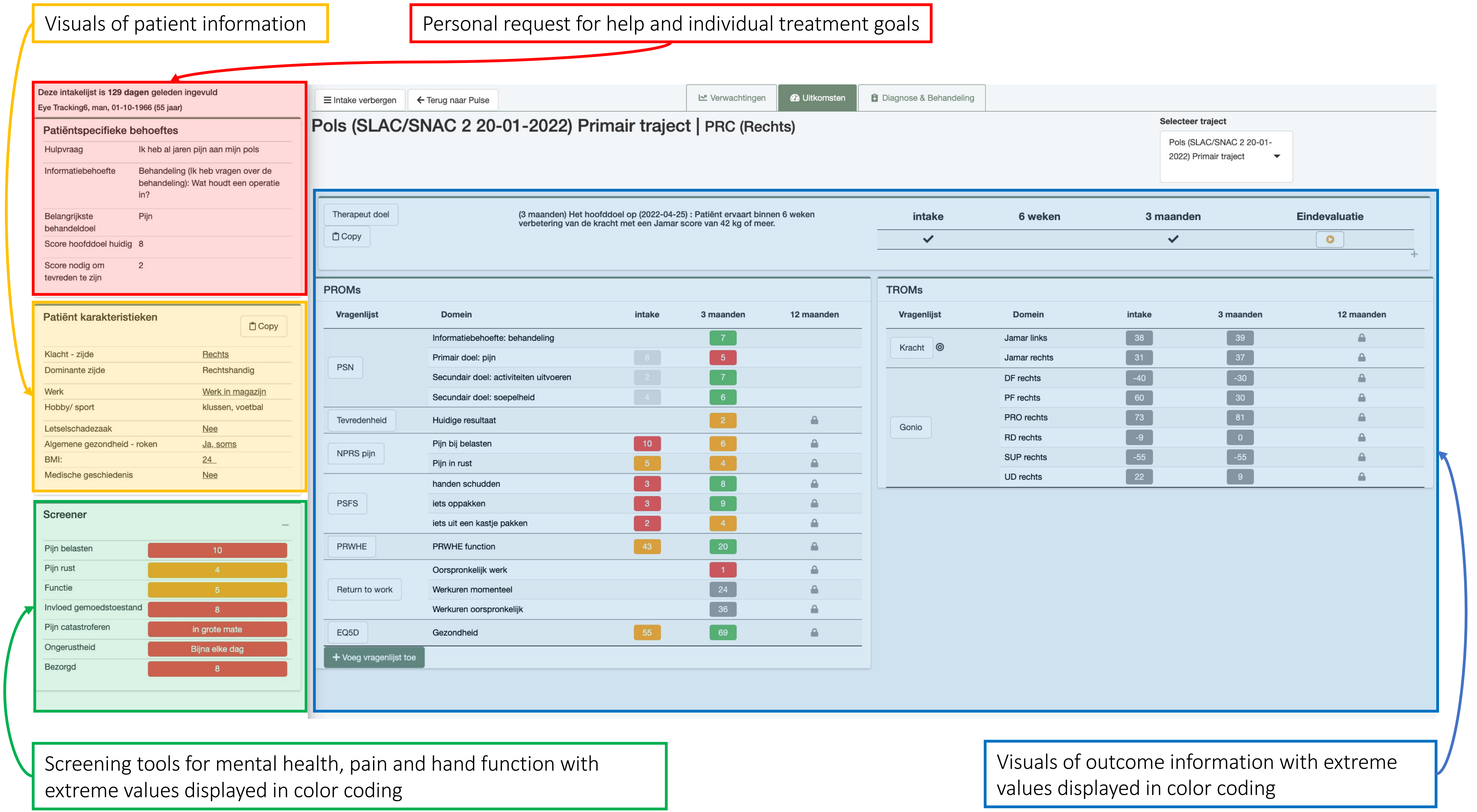
Visuals of patient information
Personal request for help and individual treatment goals
Deze intakelijst is 129 dagen geleden ingevuld
Eye Tracking6, man, 01-10-1966 (55 jaar)
Patiëntspecifieke behoeftes
Hulpvraag
Ik heb al jaren pijn aan mijn pols
Informatiebehoefte
Behandeling (Ik heb vragen over de behandeling): Wat houdt een operatie in?
Belangrijkste behandeldoel
Pijn
Score hoofddoel huidig
8
Score nodig om tevreden te zijn
2
Patiënt karakteristieken
Copy
Klacht - zijde
Rechts
Dominante zijde
Rechtshandig
Werk
Werk in magazijn
Hobby/ sport
klussen, voetbal
Letselschadezaak
Nee
Algemene gezondheid - roken
Ja, soms
BMI:
24
Medische geschiedenis
Nee
Screener
Pijn belasten
10
Pijn rust
4
Functie
5
Invloed gemoedstoestand
8
Pijn catastroferen
in grote mate
Ongerustheid
Bijna elke dag
Bezorgd
8
Intake verbergen
Terug naar Pulse
Verwachtingen
Uitkomsten
Diagnose & Behandeling
Pols (SLAC/SNAC 2 20-01-2022) Primair traject | PRC (Rechts)
Selecteer traject
Pols (SLAC/SNAC 2 20-01-2022) Primair traject
Therapeut doel
Copy
(3 maanden) Het hoofddoel op (2022-04-25) : Patiënt ervaart binnen 6 weken verbetering van de kracht met een Jamar score van 42 kg of meer.
intake
6 weken
3 maanden
Eindevaluatie
PROMs
Vragenlijst
Domein
intake
3 maanden
12 maanden
PSN
Informatiebehoefte: behandeling
7
Primair doel: pijn
8
5
Secundair doel: activiteiten uitvoeren
2
7
Secundair doel: soepelheid
4
6
Tevredenheid
Huidige resultaat
2
NPRS pijn
Pijn bij belasten
10
6
Pijn in rust
5
4
PSFS
handen schudden
3
8
iets oppakken
3
9
iets uit een kastje pakken
2
4
PRWHE
PRWHE function
43
20
Return to work
Oorspronkelijk werk
1
Werkuren momenteel
24
Werkuren oorspronkelijk
36
EQ5D
Gezondheid
55
69
Voeg vragenlijst toe
TROMs
Vragenlijst
Domein
intake
3 maanden
12 maanden
Kracht
Jamar links
38
39
Jamar rechts
31
37
Gonio
DF rechts
-40
-30
PF rechts
60
30
PRO rechts
73
81
RD rechts
-9
0
SUP rechts
-55
-55
UD rechts
22
9
Screening tools for mental health, pain and hand function with extreme values displayed in color coding
Visuals of outcome information with extreme values displayed in color coding

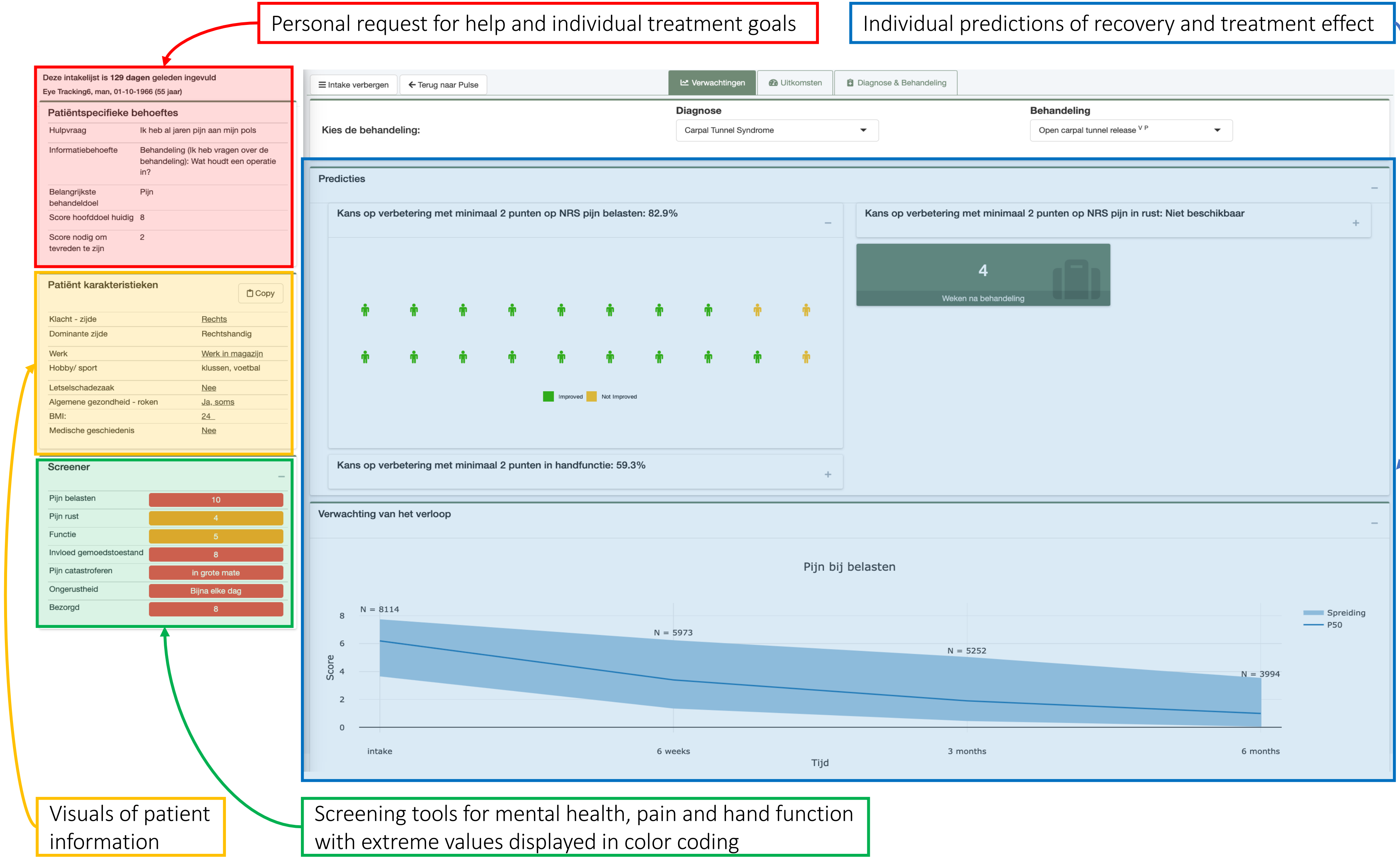
Personal request for help and individual treatment goals
Individual predictions of recovery and treatment effect
Deze intakelijst is 129 dagen geleden ingevuld
Eye Tracking6, man, 01-10-1966 (55 jaar)
Patiëntspecifieke behoeftes
Hulpvraag
Ik heb al jaren pijn aan mijn pols
Informatiebehoefte
Behandeling (Ik heb vragen over de behandeling): Wat houdt een operatie in?
Belangrijkste behandeldoel
Pijn
Score hoofddoel huidig
8
Score nodig om tevreden te zijn
2
Patiënt karakteristieken
Copy
Klacht - zijde
Rechts
Dominante zijde
Rechtshandig
Werk
Werk in magazijn
Hobby/ sport
klussen, voetbal
Letselschadezaak
Nee
Algemene gezondheid - roken
Ja, soms
BMI:
24
Medische geschiedenis
Nee
Screener
Pijn belasten
10
Pijn rust
4
Functie
5
Invloed gemoedstoestand
8
Pijn catastroferen
in grote mate
Ongerustheid
Bijna elke dag
Bezorgd
8
Intake verbergen
Terug naar Pulse
Verwachtingen
Uitkomsten
Diagnose & Behandeling
Kies de behandeling:
Diagnose
Carpal Tunnel Syndrome
Behandeling
Open carpal tunnel release V P
Predicties
Kans op verbetering met minimaal 2 punten op NRS pijn belasten: 82.9%
Improved
Not Improved
Kans op verbetering met minimaal 2 punten op NRS pijn in rust: Niet beschikbaar
4
Weken na behandeling
Kans op verbetering met minimaal 2 punten in handfunctie: 59.3%
Verwachting van het verloop
Pijn bij belasten
N = 8114
N = 5973
N = 5252
N = 3994
Spreiding
P50
Score
8
6
4
2
0
intake
6 weeks
3 months
6 months
Tijd
Visuals of patient information
Screening tools for mental health, pain and hand function with extreme values displayed in color coding
